# Gaussian Optimality for Derivatives of Differential Entropy Using Linear Matrix Inequalities †

**DOI:** 10.3390/e20030182

**Published:** 2018-03-09

**Authors:** Xiaobing Zhang, Venkat Anantharam, Yanlin Geng

**Affiliations:** 1 Shanghai Institute of Microsystem and Information Technology, Chinese Academy of Sciences, Shanghai 200050, China; 2 School of Information Science and Technology, ShanghaiTech University, Shanghai 201210, China; 3 University of Chinese Academy of Sciences, Beijing 100049, China; 4 Department of Electrical Engineering and Computer Sciences (EECS), University of California, Berkeley, CA 94720-1234, USA; 5 Shanghai Institute of Fog Computing Technology, Shanghai Tech University, Shanghai 201210, China

**Keywords:** differential entropy, entropy power, log-concavity, linear matrix inequality, Gaussian optimality

## Abstract

Let *Z* be a standard Gaussian random variable, *X* be independent of *Z*, and *t* be a strictly positive scalar. For the derivatives in *t* of the differential entropy of $X+\sqrt{t}Z$, McKean noticed that Gaussian *X* achieves the extreme for the first and second derivatives, among distributions with a fixed variance, and he conjectured that this holds for general orders of derivatives. This conjecture implies that the signs of the derivatives alternate. Recently, Cheng and Geng proved that this alternation holds for the first four orders. In this work, we employ the technique of linear matrix inequalities to show that: firstly, Cheng and Geng’s method may not generalize to higher orders; secondly, when the probability density function of $X+\sqrt{t}Z$ is log-concave, McKean’s conjecture holds for orders up to at least five. As a corollary, we also recover Toscani’s result on the sign of the third derivative of the entropy power of $X+\sqrt{t}Z$, using a much simpler argument.

## 1. Introduction

For a general continuous random variable *X* with probability density function f(x), its differential entropy is defined as

$$\begin{array}{c} h\left(X\right)=-\int_{-\infty }^{+\infty }f\left(x\right)\ln f\left(x\right)dx, \end{array}$$

given that the above integral exists. In [1], Shannon considered the entropy power $N\left(X\right)=\frac{1}{2\pi e}e^{2h(X)}$, and introduced the celebrated Entropy Power Inequality (EPI):

$$\begin{array}{c} e^{2h\left(X+Y\right)}\geq e^{2h(X)}+e^{2h(Y)}, \end{array}$$

where *X* and *Y* are independent, and equality holds if and only if *X* and *Y* are Gaussian. This inequality is nontrivial and was rigorously proved later by Stam [2].

**Remark** **1.** This is the full version of a conference paper submitted to ISIT 2018 [3].

There have been numerous generalizations of the EPI. In [4], Costa considered the case where *X* is perturbed by an independent standard Gaussian *Z*, and showed that $N(X+\sqrt{t}Z)$ is concave in *t* for t>0:

$$\begin{array}{c} \frac{d^{2}}{dt^{2}}N\left(X+\sqrt{t}Z\right)\leq 0,\;t>0. \end{array}$$

Toscani [5] further showed that $\frac{d^{3}}{dt^{3}}N\left(X+\sqrt{t}Z\right)\geq 0$, under the condition that the probability density function of $X+\sqrt{t}Z$ is log-concave.

Later, Villani [6] simplified Costa’s proof by directly studying the second derivative in *t* of the differential entropy instead of the second derivative of the entropy power. In the proof, it was noticed [6,7,8] that the signs of the first two derivatives of $h(X+\sqrt{t}Z)$ alternate. Along this line, Cheng and Geng [9] showed that this alternation holds for the first four derivatives, and they made the following conjecture that the alternation is true for general orders of derivatives.

**Conjecture** **1** ([9]). *The derivatives of the differential entropy $h(X+\sqrt{t}Z)$ satisfy ${\left(-1\right)}^{n-1}\times \frac{d^{n}}{dt^{n}}h\left(X+\sqrt{t}Z\right)\geq 0$ for t>0 and n≥1.*

According to Equation (3) in Lemma 2 and the comments, $2\times \frac{d}{dt}h\left(X+\sqrt{t}Z\right)$ is the Fisher information $J(X+\sqrt{t}Z)$. The above conjecture is equivalent to hypothesizing that the Fisher information of $X+\sqrt{t}Z$ is completely monotone, thus admitting a very simple characterization using the Laplace Transform [10]: there exists a finite Borel measure μ(·) such that

(1) $$\begin{array}{c} J\left(X+\sqrt{t}Z\right)=\int_{0}^{+\infty }e^{-\lambda t}\mu \left(d\lambda \right). \end{array}$$

Back in 1966, McKean [7] also studied the derivatives in *t* of $h(X+\sqrt{t}Z)$, and noticed that Gaussian *X* achieves the minimum of $\frac{d}{dt}h\left(X+\sqrt{t}Z\right)$ and $-\frac{d^{2}}{dt^{2}}h\left(X+\sqrt{t}Z\right)$, subject to $\mathrm{Var}\left(X\right)=\sigma^{2}$. Then, McKean implicitly made the following conjecture that Gaussian optimality holds generally:

**Conjecture** **2** ([7]). *Subject to $\mathrm{Var}\left(X\right)=\sigma^{2}$, Gaussian X with variance $\sigma^{2}$ achieves the minimum of ${\left(-1\right)}^{n-1}\times \frac{d^{n}}{dt^{n}}h\left(X+\sqrt{t}Z\right)$ for t>0 and n≥1.*

Notice that, if $X_{G}$ is Gaussian with variance $\sigma^{2}$, by routine calculation,

(2) $$\begin{array}{cc} 2h(X_{G}+\sqrt{t}Z) & =\ln2\pi e(\sigma^{2}+t),\\ {\left(-1\right)}^{n-1}\times 2\frac{d^{n}}{dt^{n}}h\left(X_{G}+\sqrt{t}Z\right) & =\left(n-1\right)!\times {\left(\sigma^{2}+t\right)}^{-n}>0. \end{array}$$

Hence, McKean’s conjecture implies the one by Cheng and Geng.

Compared with the progress made by Cheng and Geng [9] on Conjecture 1, there has been little progress on Conjecture 2. Most of the existing results are on the second derivative of the differential entropy (or the mutual information), and on generalizing the EPI to other settings. For example: Guo et al. [11] represents the derivatives in the signal-to-noise ratio of the mutual information in terms of the minimum mean-square estimation error, building on de Bruijn’s identity [2]; Wibisono and Jog [12] study the mutual information along the density flow defined by the heat equation and show that it is a convex function of time if the initial distribution is log-concave; Wang and Madiman [13] recover the proof of the EPI via rearrangements; Courtade [14] generalizes Costa’s EPI to non-Gaussian additive perturbations; and König and Smith [15] propose a quantum version of the EPI.

In this paper, we work on Conjecture 2. The main results are to show that Conjecture 2 holds for higher orders up to at least five under the log-concavity condition, and the introduction of the technique of linear matrix inequalities.

The paper is organized as follows: in Section 2, we obtain the formulae for the derivatives of the differential entropy $h(X+\sqrt{t}Z)$ (Theorem 1) and show that McKean’s conjecture holds for higher orders up to at least five under the log-concavity condition (Corollary 1). As a corollary, we recover Toscani’s result [5] on the third derivative of the entropy power, using the Cauchy–Schwartz inequality, which is much simpler. In Section 3, we introduce the linear matrix inequality approach, and transform the above two conjectures to the feasibility check of semidefinite programming problems. With this approach, we can easily obtain the coefficients in Theorem 1. Then, we show that the direct generalization of the method by Cheng and Geng might not work for orders higher than four for proving Conjecture 1. In Section 4, we prove the main theorem of Section 2.

## 2. Main Results

We first introduce the notation that is used throughout this paper. When the functions are single-variate, we use $\frac{d\;\cdot }{d\;\cdot }$ for its derivative. For the multi-variate cases, we use $\frac{\partial \;\cdot }{\partial \;\cdot }$ for the partial derivative. To simplify the notation, for the derivatives of a general single-variate function g(y), we also use $g^{\prime }\left(y\right)$, $g^{\prime \prime }\left(y\right)$ and $g^{\prime \prime \prime }\left(y\right)$ to represent the first, second and third derivatives, respectively; and $g^{\left(n\right)}\left(y\right)$ denotes the *n*-th derivative for n≥1.

In the rest of the paper, let *Z* be a standard Gaussian random variable, and *X* be independent of *Z*. Denote

$$Y_{t}:=X+\sqrt{t}Z,\;t>0.$$

According to [4,16], $Y_{t}$ has nice properties: The probability density function f(y,t) of $Y_{t}$ exists, is strictly positive and infinitely differentiable; The differential entropy $h\left(Y_{t}\right)$ exists. Denote

$$\begin{array}{cc} f_{n}:= & \frac{\partial^{n}}{\partial y^{n}}f\left(y,t\right),\\ T_{n}:= & \frac{\partial^{n}}{\partial y^{n}}\ln f\left(y,t\right),\;n=0,1,2,\ldots , \end{array}$$

where it is understood that $f_{n}$ and $T_{n}$ are functions of (y,t). We also present some properties of f(y,t) in the following lemma. The proof can be found in, say, [2,16] and Propositions 1 and 2 in [9].

**Lemma** **1.** *For t>0, the probability density function f(y,t) satisfies the following properties:*

*(1)*  The heat equation holds: $\frac{\partial }{\partial t}f=\frac{1}{2}\frac{\partial^{2}}{\partial y^{2}}f$.
*(2)*  $\lim_{|y|\to \infty }f_{n}=0$, ∀n≥0, ∀t>0.
*(3)*  The expectation of the product of the $T_{i}$, $E[\prod_{i}T_{i}]$ exists, and $\lim_{|y|\to \infty }f\prod_{i}T_{i}=0$, ∀t>0.

In Lemma 1, part (3), in writing $E[\prod_{i}T_{i}],$ we think of each $T_{i}$ as a function of $\left(Y_{t},t\right)$.

Notice that, given *X* and *Z*, the differential entropy $h(X+\sqrt{t}Z)$ is a function of *t*. The formulae for the first and second derivatives of $h(X+\sqrt{t}Z)$ are presented in the following lemma. According to Stam [2], the first equality is due to de Bruijn, and the right-hand side is actually the Fisher information (page 671 of [17]); the second one is due to McKean [7], Toscani [8] and Villani [6]; the Gaussian optimality is due to McKean [7].

**Lemma** **2.** *For the first and second derivatives of the differential entropy $h(X+\sqrt{t}Z)$, the following expressions hold for t>0:*

(3) $$\begin{array}{cc} 2h^{\prime }\left(X+\sqrt{t}Z\right) & =E\left[{\left(\frac{f_{1}}{f}\right)}^{2}\right], \end{array}$$

(4) $$\begin{array}{cc} -2h^{\prime \prime }\left(X+\sqrt{t}Z\right) & =E\left[{\left(\frac{f_{2}}{f}-\frac{f_{1}^{2}}{f^{2}}\right)}^{2}\right]. \end{array}$$

Subject to $\mathrm{Var}X=\sigma^{2}$, Gaussian X with variance $\sigma^{2}$ minimizes $h^{\prime }\left(X+\sqrt{t}Z\right)$ and $-h^{\prime \prime }\left(X+\sqrt{t}Z\right)$.

By standard manipulations, one has

(5) $$\begin{array}{c} T_{1}=\frac{f_{1}}{f},\;T_{2}=\frac{f_{2}}{f}-\frac{f_{1}^{2}}{f^{2}}. \end{array}$$

Thus, it is straightforward to rewrite the derivatives as

(6) $$\begin{array}{cc} 2h^{\prime }\left(X+\sqrt{t}Z\right) & =E\left[T_{1}^{2}\right], \end{array}$$

(7) $$\begin{array}{cc} -2h^{\prime \prime }\left(X+\sqrt{t}Z\right) & =E\left[T_{2}^{2}\right]. \end{array}$$

For the third and fourth derivatives, one can refer to Theorems 1 and 2 in [9], where they were represented by the $f_{i}$. Notice that these representations are not unique, and the ones in [9] are sufficient for identifying the signs. Instead, in Theorem 1, we use the $T_{i}$, and this will facilitate our proof of the Gaussian optimality in Corollary 1.

**Theorem** **1.** *For t>0, the derivatives of the differential entropy $h(X+\sqrt{t}Z)$ can be expressed as:*

(8) $$\begin{array}{cc} 2h^{\left(3\right)}\left(X+\sqrt{t}Z\right) & =E\left[T_{3}^{2}-2T_{2}^{3}\right], \end{array}$$

(9) $$\begin{array}{cc} -2h^{\left(4\right)}\left(X+\sqrt{t}Z\right) & =E\left[T_{4}^{2}+6T_{2}^{4}-12T_{3}^{2}T_{2}\right], \end{array}$$

(10) $$\begin{array}{cc} 2h^{\left(5\right)}\left(X+\sqrt{t}Z\right) & =E\left[T_{5}^{2}-24T_{2}^{5}-8T_{4}^{2}T_{2}-6T_{3}^{2}T_{2}T_{1}^{2}+12T_{5}T_{3}T_{2}+114T_{3}^{2}T_{2}^{2}\right]. \end{array}$$

The proof to this theorem is left to Section 4. The existence of such expressions and how to obtain the coefficients are left to Section 3, where the method of linear matrix inequalities is introduced.

### Log-Concave Case

Lemma 2 already ensures the optimality of Gaussians, subject to $\mathrm{Var}\left(X\right)=\sigma^{2}$, for the first and second derivatives. For higher ones, we do not know if we can show the optimality based on the expressions in Theorem 1. Here, we impose the constraint of log-concavity on f(y,t) and summarize the results in Corollaries 1–3.

A nonnegative function f(·) is logarithmically concave (or log-concave for short) if its domain is convex and it satisfies the inequality

$$\begin{array}{c} f\left(\theta x+(1-\theta )y\right)\geq f{\left(x\right)}^{\theta }f{\left(y\right)}^{1-\theta } \end{array}$$

for all x,y in the domain and 0<θ<1. If *f* is strictly positive, this is equivalent to saying that the logarithm of the function is concave (Section 2.5 of [18]). In our case, assuming that f(y,t) is log-concave in *y* is equivalent to $T_{2}\leq 0$.

Examples of log-concave distributions include the Gaussian, exponential, Laplace, and the Gamma with parameter larger than one. Notice that, if the probability density function of *X* is log-concave, then so is that of $X+\sqrt{t}Z$ (Section 3.5.2 of [18]).

**Corollary** **1.** If the probability density function of $X+\sqrt{t}Z$ is log-concave, then, subject to $\mathrm{Var}\left(X\right)=\sigma^{2}$, Gaussian X with variance $\sigma^{2}$ achieves the minimum of ${\left(-1\right)}^{n-1}h^{\left(n\right)}\left(X+\sqrt{t}Z\right)$ for t>0 and 3≤n≤5.

**Proof.**  Let $X_{G}$ be Gaussian with mean μ and variance $\sigma^{2}$. The probability density function of $Y_{G}:=X_{G}+\sqrt{t}Z$ is

$$f\left(y_{G},t\right)=\frac{1}{\sqrt{2\pi \left(\sigma^{2}+t\right)}}\times \exp\left\{-\frac{{\left(y_{G}-\mu \right)}^{2}}{2\left(\sigma^{2}+t\right)}\right\}.$$

The key observation is that the second derivative of the logarithm in the Gaussian case is

$$\begin{array}{c} T_{2,G}:=\frac{\partial^{2}}{\partial y_{G}^{2}}\ln f\left(y_{G},t\right)=-{\left(\sigma^{2}+t\right)}^{-1}. \end{array}$$

Hence, from Equation (2), the derivatives of the differential entropy in the Gaussian case are

$${\left(-1\right)}^{n-1}\times 2h^{\left(n\right)}\left(X_{G}+\sqrt{t}Z\right)=\left(n-1\right)!\times {\left(\sigma^{2}+t\right)}^{-n}=\left(n-1\right)!\times E{\left[-T_{2,G}\right]}^{n}.$$

Now, if one can show the following chain of inequalities:

(11) $$\begin{array}{c} {\left(-1\right)}^{n-1}\times 2h^{\left(n\right)}\left(X+\sqrt{t}Z\right)\overset{\left(a\right)}{\geq }\left(n-1\right)!\times E\left[{\left(-T_{2}\right)}^{n}\right]\overset{\left(b\right)}{\geq }\left(n-1\right)!\times E{\left[-T_{2}\right]}^{n}\overset{\left(c\right)}{\geq }\left(n-1\right)!\times E{\left[-T_{2,G}\right]}^{n}, \end{array}$$

then one is done.

For inequality (b), the log-concavity condition, namely $T_{2}\leq 0$, suffices.

For inequality (c), it suffices to show that $E\left[-T_{2}\right]\geq E\left[-T_{2,G}\right]\geq 0$. This can be proved using Lemma 2: Notice that

$$E\left[\frac{f_{2}}{f}\right]=\int_{-\infty }^{+\infty }f_{2}\left(y,t\right)dy=\int_{-\infty }^{+\infty }df_{1}\left(y,t\right)=f_{1}\left(y,t\right)\left|{}_{-\infty }^{+\infty }\right.=0-0,$$

where the last equality is due to Lemma 1.

Now, from Equation (5),

(12) $$\begin{array}{cc} E\left[-T_{2}\right] & =E\left[-\frac{f_{2}}{f}+\frac{f_{1}^{2}}{f^{2}}\right]=E\left[\frac{f_{1}^{2}}{f^{2}}\right]. \end{array}$$

Combining this with Lemma 2, one has

$$E\left[-T_{2}\right]=2h^{\prime }\left(X+\sqrt{t}Z\right)\geq 2h^{\prime }\left(X_{G}+\sqrt{t}Z\right)=E\left[-T_{2,G}\right].$$

This part is finished by noticing that $E\left[-T_{2,G}\right]>0$ from Equation (2).

For inequality (a), we show each case of *n* using Theorem 1 and the condition $T_{2}\leq 0$. For n=3,

$$2h^{\left(3\right)}\left(X+\sqrt{t}Z\right)=E\left[T_{3}^{2}-2T_{2}^{3}\right]\geq E\left[-2T_{2}^{3}\right]=\left(n-1\right)!\times E\left[{\left(-T_{2}\right)}^{n}\right],\;n=3.$$

For n=4,

$$-2h^{\left(4\right)}\left(X+\sqrt{t}Z\right)=E\left[T_{4}^{2}+6T_{2}^{4}-12T_{3}^{2}T_{2}\right]\geq E\left[6T_{2}^{4}\right]=\left(n-1\right)!\times E\left[{\left(-T_{2}\right)}^{n}\right],\;n=4,$$

where the inequality is due to $T_{2}\leq 0$, thus $E[-12T_{3}^{2}T_{2}]\geq 0$. For n=5,

$$\begin{array}{cc} 2h^{\left(5\right)}\left(X+\sqrt{t}Z\right) & =E\left[T_{5}^{2}-24T_{2}^{5}-8T_{4}^{2}T_{2}-6T_{3}^{2}T_{2}T_{1}^{2}+12T_{5}T_{3}T_{2}+114T_{3}^{2}T_{2}^{2}\right]\\ & =E\left[{\left(T_{5}+6T_{3}T_{2}\right)}^{2}-24T_{2}^{5}-8T_{4}^{2}T_{2}-6T_{3}^{2}T_{2}T_{1}^{2}+78T_{3}^{2}T_{2}^{2}\right]\\ & \geq E\left[-24T_{2}^{5}\right]\\ & =\left(n-1\right)!\times E\left[{\left(-T_{2}\right)}^{n}\right],\;n=5. \end{array}$$

Now, the proof is finished. ☐

The following corollary deals with the fifth-order case in [9], under the log-concavity assumption. The proof follows directly from Corollary 1 and Equation (2).

**Corollary** **2.** If the probability density function of $X+\sqrt{t}Z$ is log-concave, then the fifth derivative of the differential entropy is strictly positive: $h^{\left(5\right)}\left(X+\sqrt{t}Z\right)>0$ for t>0.

Regarding the entropy power, it is already known that $N^{\prime }\left(X+\sqrt{t}Z\right)\geq 0$ from the connection with Fisher information, and $N^{\prime \prime }\left(X+\sqrt{t}Z\right)\leq 0$ according to [4]. For the third derivative, Toscani showed that $N^{\left(3\right)}\left(X+\sqrt{t}Z\right)\geq 0$, under the log-concavity assumption. Here, we simplify Toscani’s proof, using a Cauchy–Schwartz argument.

**Corollary** **3.** If the probability density function of $X+\sqrt{t}Z$ is log-concave, then the third derivative of the entropy power is nonnegative: $N^{\left(3\right)}\left(X+\sqrt{t}Z\right)\geq 0$ for t>0.

**Proof.**  For brevity, let $h^{\prime }:=h^{\prime }\left(X+\sqrt{t}Z\right)$, and, similarly, we omit the arguments for higher orders. Routine manipulations yield that

$$\begin{array}{c} N^{\left(3\right)}\left(X+\sqrt{t}Z\right)=\frac{d^{3}}{dt^{3}}\frac{1}{2\pi e}e^{2h(X+\sqrt{t}Z)}=\frac{1}{2\pi e}e^{2h(X+\sqrt{t}Z)}\left[{\left(2h^{\prime }\right)}^{3}+3\times 2h^{\prime }\times 2h^{\prime \prime }+2h^{\prime \prime \prime }\right]. \end{array}$$

Thus, it suffices to show ${\left(2h^{\prime }\right)}^{3}+3\times 2h^{\prime }\times 2h^{\prime \prime }+2h^{\prime \prime \prime }\geq 0$. First, we express $h^{\prime }$, $h^{\prime \prime }$ and $h^{\prime \prime \prime }$ in the form of the $T_{i}$: according to Lemma 2 and Equation (12), $2h^{\prime }=E\left[-T_{2}\right]$; from Equation (7), $2h^{\prime \prime }=-E\left[T_{2}^{2}\right]$; copying from Equation (8), $2h^{\prime \prime \prime }=E\left[T_{3}^{2}-2T_{2}^{3}\right]$.

Also notice that, from Lemma 2, $2h^{\prime }\left(X+\sqrt{t}Z\right)\geq 2h^{\prime }\left(X_{G}+\sqrt{t}Z\right)={\left(\sigma_{X}^{2}+t\right)}^{-1}>0$. Hence, $E[-T_{2}]>0$ (it cannot be zero). Now, under the log-concavity condition, namely $T_{2}\leq 0$, from the Cauchy–Schwartz inequality for random variables, we have:

$$\begin{array}{c} E\left[-T_{2}\right]E\left[-T_{2}^{3}\right]=E\left[{\sqrt{-T_{2}}}^{2}\right]E\left[{\sqrt{-T_{2}^{3}}}^{2}\right]\geq E{\left[\sqrt{-T_{2}}\sqrt{-T_{2}^{3}}\right]}^{2}=E{\left[T_{2}^{2}\right]}^{2}. \end{array}$$

Thus, we have

$$\begin{array}{cc} {\left(2h^{\prime }\right)}^{3}+3\times 2h^{\prime }\times 2h^{\prime \prime }+2h^{\prime \prime \prime } & =E{\left[-T_{2}\right]}^{3}-3\times E\left[-T_{2}\right]\times E\left[T_{2}^{2}\right]+E\left[T_{3}^{2}-2T_{2}^{3}\right]\\ & \geq E{\left[-T_{2}\right]}^{3}-3\times E\left[-T_{2}\right]\times E\left[T_{2}^{2}\right]+E\left[-2T_{2}^{3}\right]\\ & ={\left(E[-T_{2}]\right)}^{-1}\left(E{\left[-T_{2}\right]}^{4}-3\times E{\left[-T_{2}\right]}^{2}\times E\left[T_{2}^{2}\right]+2E\left[-T_{2}\right]E\left[-T_{2}^{3}\right]\right)\\ & \geq {\left(E[-T_{2}]\right)}^{-1}\left(E{\left[-T_{2}\right]}^{4}-3\times E{\left[-T_{2}\right]}^{2}\times E\left[T_{2}^{2}\right]+2E{\left[T_{2}^{2}\right]}^{2}\right)\\ & ={\left(E[-T_{2}]\right)}^{-1}\left(E\left[T_{2}^{2}\right]-E{\left[-T_{2}\right]}^{2}\right)\left(2E\left[T_{2}^{2}\right]-E{\left[-T_{2}\right]}^{2}\right). \end{array}$$

The proof is finished by noticing that $E\left[T_{2}^{2}\right]\geq E{\left[-T_{2}\right]}^{2}\geq 0$, which implies that the right-hand side is nonnegative. ☐

## 3. Linear Matrix Inequalities

In this section, we introduce the method of linear matrix inequalities (LMI), and transform the proof of Conjectures 1 and 2 to the feasibility problem of LMI. This transformation also enables us to find the right coefficients in Theorem 1.

Recall that, in [9], the authors first obtained the fourth derivative as the following (Equation (27) in [9])

(13) $$\begin{array}{c} 2h^{\left(4\right)}\left(X+\sqrt{t}Z\right)=E\left[-\frac{f_{4}^{2}}{f^{2}}-\frac{4f_{2}f_{3}^{2}}{f^{3}}+\frac{4f_{1}^{2}f_{3}^{2}}{f^{4}}-\frac{3f_{2}^{4}}{f^{4}}+\frac{24f_{1}^{2}f_{2}^{3}}{f^{5}}-\frac{36f_{1}^{4}f_{2}^{2}}{f^{6}}+\frac{90f_{1}^{8}}{7f^{8}}\right]. \end{array}$$

Then, with some equalities (from integration by parts), they showed this derivative can be expressed as the negative of a sum of squares (Theorem 2 in [9]):

(14) $$\begin{array}{cc} 2h^{\left(4\right)}\left(X+\sqrt{t}Z\right) & =-E\left[{\left(\frac{f_{4}}{f}-\frac{6}{5}\frac{f_{1}f_{3}}{f^{2}}-\frac{7}{10}\frac{f_{2}^{2}}{f^{2}}+\frac{8}{5}\frac{f_{1}^{2}f_{2}}{f^{3}}-\frac{1}{2}\frac{f_{1}^{4}}{f^{4}}\right)}^{2}+{\left(\frac{2}{5}\frac{f_{1}f_{3}}{f^{2}}-\frac{1}{3}\frac{f_{1}^{2}f_{2}}{f^{3}}+\frac{9}{100}\frac{f_{1}^{4}}{f^{4}}\right)}^{2}\right.\\ & \left.+{\left(-\frac{4}{100}\frac{f_{1}^{2}f_{2}}{f^{3}}+\frac{4}{100}\frac{f_{1}^{4}}{f^{4}}\right)}^{2}+\frac{1}{300}\frac{f_{2}^{4}}{f^{4}}+\frac{56}{90,000}\frac{f_{1}^{4}f_{2}^{2}}{f^{6}}+\frac{13}{70,000}\frac{f_{1}^{8}}{f^{8}}\right]. \end{array}$$

Hence, the fourth derivative is nonpositive. The sum of squares has a natural connection with positive semidefinite matrices. The right-hand side of Equation (14) can be written as $-E[u^{T}Fu]$, where *u* is the column vector with coordinates $u=(f_{4}/f,\;\;f_{1}f_{3}/f^{2},\;\;f_{2}^{2}/f^{2},\;\;f_{1}^{2}f_{2}/f^{3},\;\;f_{1}^{4}/f^{4})$ and *F* is a positive semidefinite matrix. Thus, the method in [9] is actually to verify the existence of a suitable positive semidefinite matrix *F*. This can be cast as the feasibility of a linear matrix inequality.

A linear matrix inequality (Chapter 2 of [18]) has the form

(15) $$\begin{array}{c} F\left(x,y\right):=F_{0}+\sum_{i=1}^{I}x_{i}F_{i}+\sum_{j=1}^{J}y_{j}G_{j}⪰0, \end{array}$$

where the m×m symmetric matrices $F_{0}$, $F_{i}$, $G_{j}$, i=1,…,I, j=1,…,J are given, variables $x_{i}$ are real and $y_{j}^{\prime }$ are nonnegative, and the notation F(x,y)⪰0 means F(x,y) is positive semidefinite. The feasibility problem refers to identifying if there exists a set of $x_{i}$ and $y_{j}$ such that F(x,y) is positive semidefinite.

To reformulate the method used by Cheng and Geng [9] as an LMI feasibility problem, using the fourth derivative as an illustrative example, the main idea is: first, transform the original expression of the derivative to the form

$$-2h^{\left(4\right)}\left(X+\sqrt{t}Z\right)=E\left[u^{T}F_{0}u\right].$$

Then, transform the equalities resulting from integration by parts to the form

$$0=E[u^{T}F_{i}u],\;i=1,2,\ldots ,I.$$

Finally, try to find a set of variables $x_{i}$ such that $F_{0}+\sum_{i}x_{i}F_{i}⪰0$, which is sufficient to show that

$$-2h^{\left(4\right)}\left(X+\sqrt{t}Z\right)=E\left[u^{T}F_{0}u\right]=E\left[u^{T}\left(F_{0}+\sum_{i}x_{i}F_{i}\right)u\right]\geq 0.$$

One can notice that there is no matrix $G_{j}$ in the above statement. This is mainly because only equalities were available in [9]. When one imposes inequality constraints, for example $T_{2}\leq 0$, as in this paper, then one will be able to construct matrices $G_{j}$.

Before we proceed to introduce the details on constructing those matrices, the following observations are clear regarding $u=(f_{4}/f,\;\;f_{1}f_{3}/f^{2},\;\;f_{2}^{2}/f^{2},\;\;f_{1}^{2}f_{2}/f^{3},\;\;f_{1}^{4}/f^{4})$ and the fourth derivative $2h^{\left(4\right)}\left(X+\sqrt{t}Z\right)$ (see Equation (13)): (a) the sum-order of derivatives for each entry of *u* is four, for example, the sum-order of $f_{1}^{2}f_{2}/f^{3}$ is 1×2+2=4; (b) the highest order of a single term in the entries of *u* is four, namely $f_{4}/f$; (c) the sum-order of each entry in the fourth derivative is eight, which is twice that of *u*.

In the following, we take the fourth derivative as an example, and show how to construct these matrices $F_{0}$ (Section 3.3), $F_{i}$ (Section 3.1 and Section 3.2), and $G_{j}$ (Section 3.4). We decide to use the $T_{k}$ as the entries of *u*, instead of the $f_{k}$, the motivation for which is clear from the proof of Corollary 1 and the desire to exploit the assumption $T_{2}\leq 0$. Based on the above observation and the expressions in Equation (5), our vector *u* is

(16) $$\begin{array}{c} u=\left(\begin{array}{ccccc} T_{4}, & T_{3}T_{1}, & T_{2}^{2}, & T_{2}T_{1}^{2}, & T_{1}^{4} \end{array}\right). \end{array}$$

Thus, $F_{0}$, $F_{i}$, $G_{j}$ are 5×5 symmetric matrices. Here, we mention that the expressions appearing as coordinates in *u* correspond to the integer partitions of four.

The organization of this section is as follows: Section 3.1, Section 3.2 and Section 3.3 deal with the sign of the fourth derivative with only equality constraints (see Conjecture 1); Section 3.4 further incorporates the inequality constraints, namely $T_{2}\leq 0$; Section 3.5 shows the manipulation for the optimality of Gaussian inputs (see Conjecture 2). In Section 3.6, we consider the sign and the Gaussian optimality for the fifth derivative.

### 3.1. Matrices $F_{i}$ from Multiple Representations

The matrices $F_{i}$ are such that $E[u^{T}F_{i}u]=0$. A trivial case is to notice that different products of the form u(i)u(j) may map to the same term, for example

$$T_{2}^{2}T_{1}^{4}=\left(T_{2}^{2}\right)\left(T_{1}^{4}\right)=\left(T_{2}T_{1}^{2}\right)\left(T_{2}T_{1}^{2}\right),\;\Rightarrow \;u\left(3\right)u\left(5\right)=u\left(4\right)u\left(4\right).$$

That is, $T_{2}^{2}T_{1}^{4}$ admits multiple representations as u(i)u(j). It is easy to construct the corresponding matrix $F_{1}$ such that $u^{T}F_{1}u=0$:

$$\begin{array}{c} F_{1}=\left[\begin{array}{ccccc} 0 & 0 & 0 & 0 & 0\\ 0 & 0 & 0 & 0 & 0\\ 0 & 0 & 0 & 0 & -1\\ 0 & 0 & 0 & 2 & 0\\ 0 & 0 & -1 & 0 & 0 \end{array}\right]. \end{array}$$

For the fourth derivative, only one term has multiple representations. There is none for the third derivative, and three for the fifth ($F_{1}$, $F_{2}$ and $F_{3}$ in Section 3.6).

### 3.2. Matrices $F_{i}$ from Integration by Parts

The equalities of the type $E[u^{T}F_{i}u]=0$ used in [9] are from integration by parts. Here, we list them one by one.

Notice that all the possible terms with sum-order eight and highest-order four are the following (the numbers in the left column are indices):

$$\begin{array}{c} \begin{array}{cccccc} 1-5: & \;T_{4}^{2}, & T_{4}T_{3}T_{1}, & T_{4}T_{2}^{2}, & T_{4}T_{2}T_{1}^{2}, & T_{4}T_{1}^{4},\\ 6-9: & \;T_{3}^{2}T_{1}^{2}, & T_{3}T_{2}^{2}T_{1}, & T_{3}T_{2}T_{1}^{3}, & T_{3}T_{1}^{5}, & \\ 10-13: & \;T_{2}^{4}, & T_{2}^{3}T_{1}^{2}, & T_{2}^{2}T_{1}^{4}, & T_{2}T_{1}^{6}, & \\ 14: & \;T_{1}^{8}, & & & & \\ 15: & \;T_{3}^{2}T_{2}. & & & & \end{array} \end{array}$$

Denote this vector as *w*.

These terms are arranged in the order such that the first (fourteen) terms can be expressed as u(i)u(j) for some *i* and *j*, while the last term(s) cannot be. We call the first terms the quadratic part $w^{qua}$, and the last term(s) the non-quadratic part $w^{non}$. Thus, $w=(w^{qua},w^{non})$.

It is not difficult to conclude that, for non-repetition, one only needs to perform integration by parts on the entries whose highest-order term is of power one. All of these entries are (eight in total):

$$\begin{array}{c} \begin{array}{cccccccc} T_{4}T_{3}T_{1}, & T_{4}T_{2}^{2}, & T_{4}T_{2}T_{1}^{2}, & T_{4}T_{1}^{4}, & T_{3}T_{2}^{2}T_{1}, & T_{3}T_{2}T_{1}^{3}, & T_{3}T_{1}^{5}, & T_{2}T_{1}^{6}. \end{array} \end{array}$$

Taking $T_{4}T_{3}T_{1}$ as an example, one can show that (Equation (18), see the end of this subsection)

$$E\left[2T_{4}T_{3}T_{1}+T_{3}^{2}T_{1}^{2}+T_{3}^{2}T_{2}\right]=0.$$

In addition, this can be written as $E[c_{1}^{T}w]=0$, where

$$c_{1}\in R^{15},\;c_{1}\left(\left[2,6,15\right]\right)=\left[2,1,1\right].$$

There are eight equalities in total and hence there are vectors $c_{1},\ldots ,c_{8}$. We put each $c_{i}$ as the *i*-th row of $C\in R^{8\times 15}$, and write those equalities as

E[Cw]=0,

where

(17) $$C=\begin{array}{cccc} & \begin{array}{ccccccccccccccc} 1 & 2 & 3 & 4 & 5 & 6 & 7 & 8 & 9 & 10 & 11 & 12 & 13 & 14 & 15 \end{array} & & \\ {}[ & \begin{array}{ccccccccccccccc} & 2 & & & & 1 & & & & & & & & & 1\\ & & 1 & & & & 1 & & & & & & & & 2\\ & & & 1 & & 1 & 2 & 1 & & & & & & & \\ & & & & 1 & & & 4 & 1 & & & & & & \\ & & & & & & 3 & & & 1 & 1 & & & & \\ & & & & & & & 2 & & & 3 & 1 & & & \\ & & & & & & & & 1 & & & 5 & 1 & & \\ & & & & & & & & & & & & 7 & 1 & \end{array} & ] & \begin{array}{c} 1\\ 2\\ 3\\ 4\\ 5\\ 6\\ 7\\ 8 \end{array} \end{array}.$$

The entries can be found in Equations (18)–(25).

We need to extract matrices *F* from these eight equalities E[Cw]=0, such that $E[u^{T}Fu]\equiv 0$. The main problem is that $c_{k}^{T}w$ may contain entries that are not expressible as u(i)u(j). In particular, for the fourth derivative, this happens when $c_{k}\left(15\right)\neq 0$. One needs to do some work to cancel these entries. The general method, which can also be used in higher-order cases, is stated below:

1. Firstly, since $w=(w^{qua},w^{non})$, we separate the blocks of *C* accordingly,
  $$\begin{array}{c} C=\left[\begin{array}{cc} C_{11} & C_{12}\\ C_{21} & 0 \end{array}\right],\;E\left[\left[\begin{array}{cc} C_{11} & C_{12}\\ C_{21} & 0 \end{array}\right]\left[\begin{array}{c} w^{qua}\\ w^{non} \end{array}\right]\right]=0. \end{array}$$
  In the above, $C_{11}\in R^{2\times 14}$, $C_{12}\in R^{2\times 1}$, $C_{21}\in R^{6\times 14}$.
2. Secondly, each row of $C_{21}$ corresponds to a symmetric matrix $F_{i}$ such that $E[u^{T}F_{i}u]\equiv 0$. In particular, for the first row of $C_{21}$, the matrix is
  $$\begin{array}{c} F_{2}=\left[\begin{array}{ccccc} 0 & 0 & 0 & 1 & 0\\ 0 & 2 & 2 & 1 & 0\\ 0 & 2 & 0 & 0 & 0\\ 1 & 1 & 0 & 0 & 0\\ 0 & 0 & 0 & 0 & 0 \end{array}\right], \end{array}$$
  such that $\frac{1}{2}u^{T}F_{2}u=T_{4}T_{2}T_{1}^{2}+T_{3}^{2}T_{1}^{2}+2T_{3}T_{2}^{2}T_{1}+T_{3}T_{2}T_{1}^{3}$. Notice a scaling of a factor of two is added here just for conciseness, and this does not affect the feasibility of (15). Similarly, the other five matrices, corresponding to the remaining rows of $C_{21}$, are
  $$\begin{array}{cc} F_{3} & =\left[\begin{array}{ccccc} 0 & 0 & 0 & 0 & 1\\ 0 & 0 & 0 & 4 & 1\\ 0 & 0 & 0 & 0 & 0\\ 0 & 4 & 0 & 0 & 0\\ 1 & 1 & 0 & 0 & 0 \end{array}\right],\;F_{4}=\left[\begin{array}{ccccc} 0 & 0 & 0 & 0 & 0\\ 0 & 0 & 3 & 0 & 0\\ 0 & 3 & 2 & 1 & 0\\ 0 & 0 & 1 & 0 & 0\\ 0 & 0 & 0 & 0 & 0 \end{array}\right],\;F_{5}=\left[\begin{array}{ccccc} 0 & 0 & 0 & 0 & 0\\ 0 & 0 & 0 & 2 & 0\\ 0 & 0 & 0 & 3 & 1\\ 0 & 2 & 3 & 0 & 0\\ 0 & 0 & 1 & 0 & 0 \end{array}\right], \end{array}$$
  $$\begin{array}{cc} F_{6} & =\left[\begin{array}{ccccc} 0 & 0 & 0 & 0 & 0\\ 0 & 0 & 0 & 0 & 1\\ 0 & 0 & 0 & 0 & 5\\ 0 & 0 & 0 & 0 & 1\\ 0 & 1 & 5 & 1 & 0 \end{array}\right],\;F_{7}=\left[\begin{array}{ccccc} 0 & 0 & 0 & 0 & 0\\ 0 & 0 & 0 & 0 & 0\\ 0 & 0 & 0 & 0 & 0\\ 0 & 0 & 0 & 0 & 7\\ 0 & 0 & 0 & 7 & 2 \end{array}\right]. \end{array}$$
3. Thirdly, for $C_{11}$ and $C_{12}$, the equalities are $E[C_{11}w^{qua}+C_{12}w^{non}]=0$. Notice $w^{non}$ cannot be expressed in a quadratic form. Supposing that we can find a column vector *z* such that $z^{T}C_{12}=0$, then $E\left[z^{T}C_{11}w^{qua}\right]=E\left[z^{T}\left(C_{11}w^{qua}+C_{12}w^{non}\right)\right]=0$. The vector *z* actually lies in the null space of $C_{12}^{T}$, and it suffices to find the basis. One way is to do the QR decomposition:
  $$C_{12}=Q\left[\begin{array}{c} U\\ 0 \end{array}\right],$$
  where *U* is upper-triangular. The null-space of $C_{12}^{T}$ has the same dimensions as the number of rows of 0 above, and a basis as the last several columns of *Q*—in particular, for the fourth derivative
  $$C_{12}=\left[\begin{array}{c} 1\\ 2 \end{array}\right]=QR=\left[\begin{array}{cc} -\frac{1}{\sqrt{5}} & -\frac{2}{\sqrt{5}}\\ -\frac{2}{\sqrt{5}} & \frac{1}{\sqrt{5}} \end{array}\right]\left[\begin{array}{c} -\sqrt{5}\\ 0 \end{array}\right].$$
  Hence, one takes *z* as the second column of *Q*, which is (after scaling for conciseness) $z^{T}=\left[\begin{array}{cc} -2, & 1 \end{array}\right]$. Then, one calculates $z^{T}C_{11}w^{qua}=-4T_{4}T_{3}T_{1}+T_{4}T_{2}^{2}-2T_{3}^{2}T_{1}^{2}+T_{3}T_{2}^{2}T_{1}$, and the corresponding matrix $F_{8}$ (scaled by a factor of two) is
  $$\begin{array}{c} F_{8}=\left[\begin{array}{ccccc} 0 & -4 & 1 & 0 & 0\\ -4 & -4 & 1 & 0 & 0\\ 1 & 1 & 0 & 0 & 0\\ 0 & 0 & 0 & 0 & 0\\ 0 & 0 & 0 & 0 & 0 \end{array}\right]. \end{array}$$

The rest of this subsection is devoted to calculating the equalities obtained from integration by parts. This is similar to that in [9], except in the form of the $T_{i}$. To begin, we need the following lemma.

**Lemma** **3.** *Let A be a linear combination of terms of products of the $T_{i}$, then, for n≥2,*

$$E\left[T_{n}A+T_{n-1}\frac{\partial }{\partial y}A+T_{n-1}T_{1}A\right]=0.$$

**Proof.**  From calculus,

$$\begin{array}{cc} E[T_{n}A] & =\int fT_{n}Ady=\int fAdT_{n-1}\\ & \overset{\left(a\right)}{=}0-\int T_{n-1}d\left(fA\right)\\ & =-\int T_{n-1}\left(f_{1}A+f\frac{\partial }{\partial y}A\right)dy\\ & \overset{\left(b\right)}{=}-\int T_{n-1}\left(fT_{1}A+f\frac{\partial }{\partial y}A\right)dy\\ & =-E\left[T_{n-1}\frac{\partial }{\partial y}A+T_{n-1}T_{1}A\right], \end{array}$$

where (a) is due to Lemma 1, and (b) is due to Equation (5). ☐

Now, using Lemma 3, one obtains the following equalities:

(18) $$\begin{array}{cc} T_{n}=T_{4},A=T_{3}T_{1},\;\Rightarrow \; & E\left[2T_{4}T_{3}T_{1}+T_{3}^{2}T_{2}+T_{3}^{2}T_{1}^{2}\right]=0, \end{array}$$

(19) $$\begin{array}{cc} T_{n}=T_{4},A=T_{2}^{2},\;\Rightarrow \; & E\left[T_{4}T_{2}^{2}+2T_{3}^{2}T_{2}+T_{3}T_{2}^{2}T_{1}\right]=0, \end{array}$$

(20) $$\begin{array}{cc} T_{n}=T_{4},A=T_{2}T_{1}^{2},\;\Rightarrow \; & E\left[T_{4}T_{2}T_{1}^{2}+T_{3}^{2}T_{1}^{2}+2T_{3}T_{2}^{2}T_{1}+T_{3}T_{2}T_{1}^{3}\right]=0, \end{array}$$

(21) $$\begin{array}{cc} T_{n}=T_{4},A=T_{1}^{4},\;\Rightarrow \; & E\left[T_{4}T_{1}^{4}+4T_{3}T_{2}T_{1}^{3}+T_{3}T_{1}^{5}\right]=0, \end{array}$$

(22) $$\begin{array}{cc} T_{n}=T_{3},A=T_{2}^{2}T_{1},\;\Rightarrow \; & E\left[3T_{3}T_{2}^{2}T_{1}+T_{2}^{4}+T_{2}^{3}T_{1}^{2}\right]=0, \end{array}$$

(23) $$\begin{array}{cc} T_{n}=T_{3},A=T_{2}T_{1}^{3},\;\Rightarrow \; & E\left[2T_{3}T_{2}T_{1}^{3}+3T_{2}^{3}T_{1}^{2}+T_{2}^{2}T_{1}^{4}\right]=0, \end{array}$$

(24) $$\begin{array}{cc} T_{n}=T_{3},A=T_{1}^{5},\;\Rightarrow \; & E\left[T_{3}T_{1}^{5}+5T_{2}^{2}T_{1}^{4}+T_{2}T_{1}^{6}\right]=0, \end{array}$$

(25) $$\begin{array}{cc} T_{n}=T_{2},A=T_{1}^{6},\;\Rightarrow \; & E\left[7T_{2}T_{1}^{6}+T_{1}^{8}\right]=0. \end{array}$$

With these equalities, matrix (17) can be constructed.

### 3.3. Matrix $F_{0}$ from the Derivative

Suppose we have already obtained the fourth derivative in the form (see Equation (30) later)

$$-2h^{\left(4\right)}\left(X+\sqrt{t}Z\right)=E\left[d^{T}w\right]=E\left[d_{1}^{T}w^{qua}+d_{2}^{T}w^{non}\right],$$

where $d_{1}\in R^{14}$, $d_{2}\in R^{1}$. Then, similar to $F_{8}$, we can find the matrix $F_{0}$ such that $-2h^{\left(4\right)}\left(X+\sqrt{t}Z\right)=E\left[u^{T}F_{0}u\right]$.

To cancel the non-quadratic term $d_{2}^{T}w^{non}$, we solve for $z_{2}^{T}C_{12}=d_{2}^{T}$ (the solution $z_{2}$ should exist, otherwise it is not possible to find a quadratic form and the LMI approach fails). Then, since $E[C_{11}w^{qua}+C_{12}w^{non}]=0$, we have

$$\begin{array}{cc} -2h^{\left(4\right)}\left(X+\sqrt{t}Z\right) & =E\left[d_{1}^{T}w^{qua}+d_{2}^{T}w^{non}\right]\\ & =E\left[d_{1}^{T}w^{qua}+d_{2}^{T}w^{non}-z_{2}^{T}\left(C_{11}w^{qua}+C_{12}w^{non}\right)\right]\\ & =E\left[\left(d_{1}^{T}-z_{2}^{T}C_{11}\right)w^{qua}\right]. \end{array}$$

Now, $F_{0}$ can be constructed from $d_{1}^{T}-z^{T}C_{11}$.

The details are as follows. First, we need to express the derivative using the entries of *w*. This can be done recursively using the following lemma.

**Lemma** **4.** *Let A be a linear combination of terms of products of the $T_{i}$. The following equalities hold:*

(26) $$\begin{array}{c} 2\frac{\partial }{\partial t}T_{n}=\frac{\partial^{n}}{\partial y^{n}}\left(T_{2}+T_{1}^{2}\right)=T_{n+2}+\sum_{k=0}^{n}C_{n}^{k}T_{k+1}T_{n-k+1},\;n\geq 0, \end{array}$$

(27) $$\begin{array}{c} \frac{d}{dt}E\left[A\right]=E\left[\frac{1}{2}\left(T_{2}+T_{1}^{2}\right)A+\frac{\partial }{\partial t}A\right], \end{array}$$

(28) $$\begin{array}{c} \frac{d}{dt}E\left[T_{n}^{2}\right]=E\left[-T_{n+1}^{2}+T_{n}\sum_{k=1}^{n-1}C_{n}^{k}T_{k+1}T_{n-k+1}\right],\;n\geq 1. \end{array}$$

The proof is left to Appendix A. Now, with Equation (7):

$$-2h^{\prime \prime }\left(X+\sqrt{t}Z\right)=E\left[T_{2}^{2}\right],$$

and Equation (28), one can easily obtain that

(29) $$\begin{array}{c} 2h^{\left(3\right)}\left(X+\sqrt{t}Z\right)=-\frac{d}{dt}E\left[T_{2}^{2}\right]=E\left[T_{3}^{2}-2T_{2}^{3}\right]. \end{array}$$

For the fourth derivative,

(30) $$\begin{array}{cc} -2h^{\left(4\right)}\left(X+\sqrt{t}Z\right) & =-\frac{d}{dt}E\left[T_{3}^{2}-2T_{2}^{3}\right]\\ & \overset{\left(a\right)}{=}-E\left[-T_{4}^{2}+T_{3}\left(3T_{2}T_{3}+3T_{3}T_{2}\right)\right]-\frac{d}{dt}E\left[-2T_{2}^{3}\right]\\ & \overset{\left(b\right)}{=}-E\left[-T_{4}^{2}+T_{3}\left(3T_{2}T_{3}+3T_{3}T_{2}\right)\right]-E\left[-\left(T_{2}+T_{1}^{2}\right)T_{2}^{3}-3T_{2}^{2}\frac{\partial }{\partial t}2T_{2}\right]\\ & \overset{\left(c\right)}{=}-E\left[-T_{4}^{2}+6T_{3}^{2}T_{2}-T_{2}^{4}-T_{2}^{3}T_{1}^{2}-3T_{2}^{2}\left(T_{4}+2T_{3}T_{1}+2T_{2}T_{2}\right)\right]\\ & =E\left[T_{4}^{2}+3T_{4}T_{2}^{2}-6T_{3}^{2}T_{2}+6T_{3}T_{2}^{2}T_{1}+7T_{2}^{4}+T_{2}^{3}T_{1}^{2}\right], \end{array}$$

where (a), (b), (c) are due to Equations (28), (27), (26), respectively.

Hence, we have the vector $d=\left(d_{1},d_{2}\right)\in R^{15}$ and its blocks $d_{1}\in R^{14}$, $d_{2}\in R^{1}$:

$$\begin{array}{c} d[1,3,7,10,11]=[1,3,6,7,1],\\ d[15]=-6. \end{array}$$

One solves for $z_{2}$ such that $z_{2}^{T}C_{12}=d_{2}^{T}$ and obtains

$$\begin{array}{c} z_{2}^{T}=\left[\begin{array}{cc} 0, & -3 \end{array}\right]. \end{array}$$

Now, $d_{1}^{T}-z_{2}^{T}C_{11}$ has nonzero entries at locations [1,3,7,10,11], with values [1,6,9,7,1], respectively. Furthermore, $F_{0}$ (scaled by a factor of two) is found as

$$\begin{array}{c} F_{0}=\left[\begin{array}{ccccc} 2 & 0 & 6 & 0 & 0\\ 0 & 0 & 9 & 0 & 0\\ 6 & 9 & 14 & 1 & 0\\ 0 & 0 & 1 & 0 & 0\\ 0 & 0 & 0 & 0 & 0 \end{array}\right]. \end{array}$$

By the end of this subsection, it is easy to see that Cheng and Geng’s method can be reformulated as identifying if there exist $x_{1},\ldots ,x_{8}\in R$ such that

$$F_{0}+\sum_{i=1}^{8}x_{i}F_{i}⪰0.$$

We use the convex optimization package [19] to identify the feasibility of the above LMI problem, and it turns out to be feasible as it should be according to Equation (14).

### 3.4. Matrices $G_{j}$ from Log-Concavity

Recall that, in [9], there is no matrix $G_{j}$, since there is no inequality constraint. In this paper, we consider the log-concave case $T_{2}\leq 0$, thus introducing inequality constraints.

For the fourth order, $T_{2}\leq 0$ actually implies that the following entries in *w* are nonpositive:

$$T_{2}^{3}T_{1}^{2},\;T_{2}T_{1}^{6},\;T_{2}T_{3}^{2}.$$

It is clear that the powers of $T_{2}$ are odd, and the others are even.

To transform these nonpositive terms into matrices $G_{j}$, the first two terms, $T_{2}^{3}T_{1}^{2}$ and $T_{2}T_{1}^{6}$ are trivial, since they can be expressed by u(i)u(j) directly:

$$\begin{array}{c} 0\geq 2E\left[T_{2}^{3}T_{1}^{2}\right]=E\left[u^{T}G_{1}u\right],\\ 0\geq 2E\left[T_{2}T_{1}^{6}\right]=E\left[u^{T}G_{2}u\right], \end{array}$$

where

$$\begin{array}{c} G_{1}=\left[\begin{array}{ccccc} 0 & 0 & 0 & 0 & 0\\ 0 & 0 & 0 & 0 & 0\\ 0 & 0 & 0 & 1 & 0\\ 0 & 0 & 1 & 0 & 0\\ 0 & 0 & 0 & 0 & 0 \end{array}\right],\;G_{2}=\left[\begin{array}{ccccc} 0 & 0 & 0 & 0 & 0\\ 0 & 0 & 0 & 0 & 0\\ 0 & 0 & 0 & 0 & 0\\ 0 & 0 & 0 & 0 & 1\\ 0 & 0 & 0 & 1 & 0 \end{array}\right]. \end{array}$$

For the term $T_{2}T_{3}^{2}$, the idea is similar to the third part in Section 3.2. One first finds $z_{3}\in R^{2}$ such that $z_{3}^{T}C_{12}w^{non}=T_{2}T_{3}^{2}$, namely $z_{3}^{T}C_{12}=1$. The solution is $z_{3}^{T}=\left[\begin{array}{cc} 0, & 1/2 \end{array}\right]$. Then,

$$\begin{array}{c} E\left[T_{2}T_{3}^{2}\right]=E\left[T_{2}T_{3}^{2}-z_{3}^{T}\left(C_{11}w^{qua}+C_{12}w^{non}\right)\right]=E\left[-z_{3}^{T}C_{11}w^{qua}\right]=E\left[-\frac{1}{2}T_{4}T_{2}^{2}-\frac{1}{2}T_{3}T_{2}^{2}T_{1}\right]. \end{array}$$

Now, it is routine to obtain

$$\begin{array}{c} 0\geq 4E\left[T_{2}T_{3}^{2}\right]=E\left[u^{T}G_{3}u\right], \end{array}$$

where

$$\begin{array}{c} G_{3}=\left[\begin{array}{ccccc} 0 & 0 & -1 & 0 & 0\\ 0 & 0 & -1 & 0 & 0\\ -1 & -1 & 0 & 0 & 0\\ 0 & 0 & 0 & 0 & 0\\ 0 & 0 & 0 & 0 & 0 \end{array}\right]. \end{array}$$

At this point, we are done with the procedure for calculating all these matrices $F_{0}$, the $F_{i}$ and the $G_{j}$. To show the negativity of the fourth derivative, it suffices to find a set of variables $x_{i}\in R$ and $y_{j}\geq 0$ such that

$$\begin{array}{c} F_{0}+\sum_{i=1}^{8}x_{i}F_{i}+\sum_{j=1}^{3}y_{j}G_{j}⪰0. \end{array}$$

**Remark** **2.** The matrix $G_{2}$ is actually redundant, since we know that $E\left[T_{2}T_{1}^{6}\right]\equiv -\frac{1}{7}E\left[T_{1}^{8}\right]\leq 0$, which is already included in the matrices $F_{i}$ (in particular, matrix $F_{7}$ in Section 3.2). Including $G_{2}$ will not affect the feasibility check.

### 3.5. Matrix ${\tilde{F}}_{0}$ for Gaussian Optimality

However, to show the optimality of the Gaussian, the above formulation is not enough. According to inequality (a) in Equation (11), it would suffice to show that

$${\left(-1\right)}^{n-1}\times 2h^{\left(n\right)}\left(X+\sqrt{t}Z\right)-\left(n-1\right)!\times E\left[{\left(-T_{2}\right)}^{n}\right]\geq 0$$

instead of ${\left(-1\right)}^{n-1}\times 2h^{\left(n\right)}\left(X+\sqrt{t}Z\right)\geq 0$. Thus, one needs to calculate the matrix $F_{0}$ such that

$${\left(-1\right)}^{n-1}\times 2h^{\left(n\right)}\left(X+\sqrt{t}Z\right)-\left(n-1\right)!\times E\left[{\left(-T_{2}\right)}^{n}\right]=E\left[u^{T}F_{0}u\right].$$

The procedure is the same as that in Section 3.3.

In particular, for the fourth derivative, since n=4 is even, we directly have the quadratic form $E\left[{\left(-T_{2}\right)}^{n}\right]=u\left(3\right)u\left(3\right)$. It is straightforward to construct the matrix ${\tilde{F}}_{0}$ (scaled by a factor of two) here

$$\begin{array}{c} {\tilde{F}}_{0}=F_{0}-\left[\begin{array}{ccccc} 0 & 0 & 0 & 0 & 0\\ 0 & 0 & 0 & 0 & 0\\ 0 & 0 & 2\times 3! & 0 & 0\\ 0 & 0 & 0 & 0 & 0\\ 0 & 0 & 0 & 0 & 0 \end{array}\right]=\left[\begin{array}{ccccc} 2 & 0 & 6 & 0 & 0\\ 0 & 0 & 9 & 0 & 0\\ 6 & 9 & 2 & 1 & 0\\ 0 & 0 & 1 & 0 & 0\\ 0 & 0 & 0 & 0 & 0 \end{array}\right], \end{array}$$

such that $E\left[u^{T}{\tilde{F}}_{0}u\right]=-4h^{\left(4\right)}\left(X+\sqrt{t}Z\right)-12E\left[T_{2}^{4}\right]$.

Now, the LMI is updated as ${\tilde{F}}_{0}+\sum_{i=1}^{8}x_{i}F_{i}+\sum_{j=1}^{3}y_{j}G_{j}⪰0.$ Again, we use the convex optimization package [19] to check the feasibility. It turns out to be feasible and the solution helps us to identify the coefficients in Equation (9).

### 3.6. Fifth Derivative

For the fifth derivative, we omit the details of the manipulations since they are routine, and just provide the matrices here. For brevity, we only list out the nonzero entries of the upper-triangular part of a symmetric matrix. These matrices (with scaling) are

$$\begin{array}{cc} F_{0}:\; & F[(1,1),(1,3),(1,5),(2,3),(2,5),(3,3),(3,4),(3,5),(3,6),(5,5),(5,6)]\\ & =[2,20,29,\frac{214}{3},\frac{49}{2},\frac{178}{3},-\frac{37}{3},58,-6,45,-\frac{1}{2}],\\ F_{1}:\; & F[(3,6),(4,5)]=[-1,1],\\ F_{2}:\; & F[(3,7),(4,6)]=[-1,1],\\ F_{3}:\; & F[(5,7),(6,6)]=[-1,2],\\ F_{4}:\; & F[(1,4),(2,2),(2,3),(2,4)]=[1,2,2,1],\\ F_{5}:\; & F[(1,6),(2,4),(2,5),(2,6)]=[1,1,3,1],\\ F_{6}:\; & F[(1,7),(2,6),(2,7)]=[1,5,1],\\ F_{7}:\; & F[(2,4),(3,4),(4,4)]=[2,3,2],\\ F_{8}:\; & F[(2,5),(3,4),(3,5),(3,6)]=[1,2,2,1],\\ F_{9}:\; & F[(2,6),(3,6),(3,7),(4,4)]=[1,4,1,2],\\ F_{10}:\; & F[(2,7),(3,7),(4,7)]=[1,6,1],\\ F_{11}:\; & F[(3,6),(5,5),(5,6)]=[3,6,1],\\ F_{12}:\; & F[(3,7),(5,6),(5,7)]=[2,5,1],\\ F_{13}:\; & F[(4,7),(5,7),(6,7)]=[1,7,1],\\ F_{14}:\; & F[(6,7),(7,7)]=[9,2],\\ F_{15}:\; & F[(1,2),(1,3),(2,2),(2,3),(3,3),(3,4)]=[6,-3,6,-5,-2,-1],\\ F_{16}:\; & F[(1,5),(2,3),(2,5),(3,3),(3,5)]=[-1,-2,-1,6,1]. \end{array}$$

For the sign of the fifth derivative, we used the convex optimization package [19] to solve the following LMI problem,

$$F_{0}+\sum_{i=1}^{16}x_{i}F_{i}⪰0,$$

but could not find a feasible solution $x_{1},\ldots ,x_{16}\in R$. This suggests to us that a direct generalization of Cheng and Geng’s method may not work for the fifth derivative.

Instead, if we consider the log-concavity constraint $T_{2}\leq 0$ and check the optimality of Gaussian inputs, then we have a new matrix ${\tilde{F}}_{0}$ (similar to Section 3.5) and several matrices $G_{j}$ as the following:

$$\begin{array}{cc} {\tilde{F}}_{0}:\; & F[(1,1),(1,3),(1,5),(2,3),(2,5),(3,3),(3,4),(3,5),(3,6),(5,5),(5,6)]\\ & =[2,20,29,\frac{214}{3},\frac{49}{2},\frac{178}{3},-\frac{37}{3},-38,-6,-3,-\frac{1}{2}],\\ G_{1}:\; & G[(3,4)]=[1],\\ G_{2}:\; & G[(5,6)]=[1],\\ G_{3}:\; & G[(6,7)]=[1],\\ G_{4}:\; & G[(1,3),(2,3),(3,3),(3,4)]=[-3,-5,-2,-1],\\ G_{5}:\; & G[(3,5),(5,5)]=[-2,-1]. \end{array}$$

Now, one would like to find $x_{1},\ldots ,x_{16}\in R$ and $y_{1},\ldots ,y_{5}\in R^{+}$ such that

$${\tilde{F}}_{0}+\sum_{i=1}^{16}x_{i}F_{i}+\sum_{i=1}^{5}y_{i}G_{i}⪰0.$$

This can be solved by the convex optimization package [19]. Again, the solution helps us to arrive at Equation (10).

## 4. Proof of Theorem 1

**Proof.**  For the third derivative, according to Equation (29), we have

$$\begin{array}{c} 2h^{\left(3\right)}\left(X+\sqrt{t}Z\right)=E\left[T_{3}^{2}-2T_{2}^{3}\right]. \end{array}$$

For the fourth derivative, according to Equation (30):

$$\begin{array}{c} -2h^{\left(4\right)}\left(X+\sqrt{t}Z\right)=E\left[T_{4}^{2}+3T_{4}T_{2}^{2}-6T_{3}^{2}T_{2}+6T_{3}T_{2}^{2}T_{1}+7T_{2}^{4}+T_{2}^{3}T_{1}^{2}\right]. \end{array}$$

Adding multiples of the left-hand sides of the equations: −3×(19)−(22), we obtain

$$\begin{array}{cc} -2h^{\left(4\right)}\left(X+\sqrt{t}Z\right) & =E\left[T_{4}^{2}+3T_{4}T_{2}^{2}-6T_{3}^{2}T_{2}+6T_{3}T_{2}^{2}T_{1}+7T_{2}^{4}+T_{2}^{3}T_{1}^{2}\right]\\ & \overset{\left(a\right)}{=}E\left[T_{4}^{2}+\left(-6T_{3}^{2}T_{2}-3T_{3}T_{2}^{2}T_{1}\right)-6T_{3}^{2}T_{2}+6T_{3}T_{2}^{2}T_{1}+7T_{2}^{4}+T_{2}^{3}T_{1}^{2}\right]\\ & =E\left[T_{4}^{2}-12T_{3}^{2}T_{2}+3T_{3}T_{2}^{2}T_{1}+7T_{2}^{4}+T_{2}^{3}T_{1}^{2}\right]\\ & \overset{\left(b\right)}{=}E\left[T_{4}^{2}-12T_{3}^{2}T_{2}+\left(-T_{2}^{4}-T_{2}^{3}T_{1}^{2}\right)+7T_{2}^{4}+T_{2}^{3}T_{1}^{2}\right]\\ & =E\left[T_{4}^{2}-12T_{3}^{2}T_{2}+6T_{2}^{4}\right], \end{array}$$

where (a) is due to Equation (19), and (b) is due to Equation (22).

For the fifth derivative,

$$\begin{array}{c} 2h^{\left(5\right)}\left(X+\sqrt{t}Z\right)=\frac{d}{dt}E\left[-T_{4}^{2}-6T_{2}^{4}+12T_{3}^{2}T_{2}\right]=\frac{d}{dt}E\left[-T_{4}^{2}\right]+\frac{d}{dt}E\left[-6T_{2}^{4}\right]+\frac{d}{dt}E\left[12T_{3}^{2}T_{2}\right]. \end{array}$$

For each term above on the right-hand side: According to Equation (28),

(31) $$\begin{array}{c} \frac{d}{dt}E\left[-T_{4}^{2}\right]=E\left[T_{5}^{2}-8T_{4}^{2}T_{2}-6T_{4}T_{3}^{2}\right]. \end{array}$$

For the second term,

(32) $$\begin{array}{cc} \frac{d}{dt}E\left[-6T_{2}^{4}\right] & \overset{\left(c\right)}{=}E\left[-3\left(T_{2}+T_{1}^{2}\right)T_{2}^{4}-12T_{2}^{3}\times \frac{\partial }{\partial t}\left(2T_{2}\right)\right]\\ & \overset{\left(d\right)}{=}E\left[-3T_{2}^{5}-3T_{2}^{4}T_{1}^{2}-12T_{2}^{3}\left(T_{4}+2T_{3}T_{1}+2T_{2}^{2}\right)\right]\\ & =E\left[-3T_{2}^{4}T_{1}^{2}-12T_{4}T_{2}^{3}-24T_{3}T_{2}^{3}T_{1}-27T_{2}^{5}\right], \end{array}$$

where (c) is due to Equation (27), and (d) is due to Equation (26). For the third term, according to Equation (27),

$$\begin{array}{c} \frac{d}{dt}E\left[12T_{3}^{2}T_{2}\right]=E\left[6\left(T_{2}+T_{1}^{2}\right)T_{3}^{2}T_{2}+\frac{\partial }{\partial t}\left(12T_{3}^{2}T_{2}\right)\right], \end{array}$$

where the last term is

$$\begin{array}{cc} \frac{\partial }{\partial t}\left(12T_{3}^{2}T_{2}\right) & =12T_{3}^{2}\frac{\partial }{\partial t}T_{2}+24T_{3}T_{2}\times \frac{\partial }{\partial t}T_{3}\\ & \overset{\left(e\right)}{=}6T_{3}^{2}\left(T_{4}+2T_{3}T_{1}+2T_{2}^{2}\right)+12T_{3}T_{2}\left(T_{5}+2T_{4}T_{1}+6T_{3}T_{2}\right)\\ & =12T_{5}T_{3}T_{2}+6T_{4}T_{3}^{2}+24T_{4}T_{3}T_{2}T_{1}+12T_{3}^{3}T_{1}+84T_{3}^{2}T_{2}^{2}, \end{array}$$

where (e) is due to Equation (26). Hence

(33) $$\begin{array}{cc} \frac{d}{dt}E\left[12T_{3}^{2}T_{2}\right] & =E\left[12T_{5}T_{3}T_{2}+6T_{4}T_{3}^{2}+24T_{4}T_{3}T_{2}T_{1}+6T_{3}^{2}T_{2}T_{1}^{2}+12T_{3}^{3}T_{1}+90T_{3}^{2}T_{2}^{2}\right]. \end{array}$$

Finally, combining Equations (31)–(33), we get

(34) $$\begin{array}{cc} 2h^{\left(5\right)}\left(X+\sqrt{t}Z\right) & =\frac{d}{dt}E\left[-T_{4}^{2}\right]+\frac{d}{dt}E\left[-6T_{2}^{4}\right]+\frac{d}{dt}E\left[12T_{3}^{2}T_{2}\right]\\ & =E\left[T_{5}^{2}-8T_{4}^{2}T_{2}-3T_{2}^{4}T_{1}^{2}-12T_{4}T_{2}^{3}-24T_{3}T_{2}^{3}T_{1}\right.\\ & \left.\;-27T_{2}^{5}+12T_{5}T_{3}T_{2}+24T_{4}T_{3}T_{2}T_{1}+6T_{3}^{2}T_{2}T_{1}^{2}+12T_{3}^{3}T_{1}+90T_{3}^{2}T_{2}^{2}\right]. \end{array}$$

To simplify Equation (34), using Lemma 3, one first obtains the following equalities:

(35) $$\begin{array}{cc} T_{n}=T_{4},\;A=T_{3}T_{2}T_{1},\;\Rightarrow \; & E\left[2T_{4}T_{3}T_{2}T_{1}+T_{3}^{3}T_{1}+T_{3}^{2}T_{2}^{2}+T_{3}^{2}T_{2}T_{1}^{2}\right]=0, \end{array}$$

(36) $$\begin{array}{cc} T_{n}=T_{3},\;A=T_{2}^{3}T_{1},\;\Rightarrow \; & E\left[4T_{3}T_{2}^{3}T_{1}+T_{2}^{5}+T_{2}^{4}T_{1}^{2}\right]=0, \end{array}$$

(37) $$\begin{array}{cc} T_{n}=T_{4},\;A=T_{2}^{3},\;\Rightarrow \; & E\left[T_{4}T_{2}^{3}+3T_{3}^{2}T_{2}^{2}+T_{3}T_{2}^{3}T_{1}\right]=0. \end{array}$$

Then, adding multiples of the left-hand sides of Equations (35)–(37), we have

$$\begin{array}{cc} 2h^{\left(5\right)}\left(X+\sqrt{t}Z\right) & =2h^{\left(5\right)}\left(X+\sqrt{t}Z\right)-12\times \left(35\right)+3\times \left(36\right)+12\times \left(37\right)\\ & =E\left[T_{5}^{2}-24T_{2}^{5}-8T_{4}^{2}T_{2}-6T_{3}^{2}T_{2}T_{1}^{2}+12T_{5}T_{3}T_{2}+114T_{3}^{2}T_{2}^{2}\right]. \end{array}$$

 ☐

## 5. Discussion

### 5.1. On the Derivatives

We are not able to say anything conclusive about the sign of the fifth derivative of the differential entropy $h(X+\sqrt{t}Z)$. If we impose the log-concavity condition, namely $T_{2}\leq 0$, then the fifth derivative is at least $4!\times E[{\left(-T_{2}\right)}^{5}]$. This motivates us to consider the following problem: Without additional constraints, what are the values $c_{5}>0$ such that

$$2h^{\left(5\right)}\left(X+\sqrt{t}Z\right)\geq c_{5}\times E\left[{\left(-T_{2}\right)}^{5}\right].$$

If one finds such a value $c_{5}$, then so long as $E[{\left(-T_{2}\right)}^{5}]\geq 0$, the sign of the fifth derivative is determined. This condition is much weaker than $T_{2}\leq 0$.

For the computational part, one only needs to construct the matrix ${\tilde{F}}_{0}$ such that $2h^{\left(5\right)}\left(X+\sqrt{t}Z\right)-c_{5}\times E\left[{\left(-T_{2}\right)}^{5}\right]=E\left[u^{T}{\tilde{F}}_{0}u\right]$, and then solve the problem (see Section 3.6 for the matrices $F_{i}$)

$${\tilde{F}}_{0}+\sum_{i=1}^{16}x_{i}F_{i}⪰0.$$

It turns out that $c_{5}=0.13$ works, while $c_{5}=0.125$ fails. The authors guess that $c_{5}\in \left[0.13,24\right]$ works, but, at the moment, can just partly confirm this with limited simulation.

Notice that the third derivative of the entropy power $N(X+\sqrt{t}Z)$ was shown to be nonnegative under the log-concavity condition [5], and we recover this in Corollary 3. We also considered the fourth derivative, but failed to obtain the sign because we were unable to apply the Cauchy–Schwartz inequality as we did for the third derivative.

### 5.2. Possible Proofs

To prove Conjecture 1, besides the method proposed in [9], we are also considering the following ways: the first one is constructive and inspired by Equation (1). Given a random variable *X*, if we can construct a proper measure μ(·) such that Equation (1) holds, then one proves Conjecture 1. However, this is difficult even when *X* is binary symmetric, which is a very simple random variable.

The second one is recursive. Suppose one can find a formula for the *n*-th derivative such that

$$\begin{array}{cc} {\left(-1\right)}^{n-1}\times h^{\left(n\right)}\left(X+\sqrt{t}Z\right) & =E[\sum_{i=1}^{k_{n}}A_{i}^{2}],\\ \frac{d}{dt}E\left[A_{i}^{2}\right] & =E[-B_{i}^{2}], \end{array}$$

then it is clear that

$$\begin{array}{cc} {\left(-1\right)}^{n}\times h^{\left(n+1\right)}\left(X+\sqrt{t}Z\right) & =E[\sum_{i=1}^{k_{n}}B_{i}^{2}]\geq 0. \end{array}$$

However, this fails for n=2 (see Equation (7) and Theorem 1). Instead, one may expect that

$$\begin{array}{cc} \frac{d}{dt}E\left[A_{i}^{2}\right] & =E[-B_{i}^{2}-C_{i}^{2}+B_{i+1}^{2}], \end{array}$$

and then

$$\begin{array}{cc} {\left(-1\right)}^{n}\times h^{\left(n+1\right)}\left(X+\sqrt{t}Z\right) & =E[-B_{1}^{2}+B_{k_{n}+1}^{2}-\sum_{i=1}^{k_{n}}C_{i}^{2}]. \end{array}$$

If further one can show that $E\left[-B_{1}^{2}+B_{k_{n}+1}^{2}\right]=E\left[-C_{k_{n}+1}^{2}\right]$ for some $C_{k_{n}+1}$, then one finishes the proof. Notice here that a clever observation is needed for this way to work.

### 5.3. Applications

The topic of Gaussian optimality has wide applications, for example in [20,21]. In this work, besides the Gaussian optimality, we also have some new observations. In [11], the derivatives in the signal-noise ratio (snr) of $I(X;\sqrt{snr}X+Z)$ are studied. In particular, the first four derivatives are obtained in the language of the minimum mean-square error (Equations (69)–(72) in Corollary 1 of [11]). However, it is not clear whether some of these derivatives are signed or not.

With some standard manipulations, it is not difficult to show that

$$\begin{array}{c} I\left(X;\sqrt{snr}X+Z\right)=h\left(\sqrt{snr}X+Z\right)-h\left(Z\right)=h\left(X+\frac{1}{\sqrt{snr}}Z\right)+\log\sqrt{snr}-\frac{1}{2}\log2\pi e. \end{array}$$

By letting $t=1/\sqrt{snr}$, one can easily connect the minimum mean-square error formulae in [11] with the signs of the derivatives of $h(X+\sqrt{t}Z)$ in *t*. The verification of Conjectures 1 and 2 would imply the bounding and extremal properties of Equations (69)–(72) in [11], and thus deepen our understanding of the minimum mean-square error estimation under the additive-Gaussian setting.

In addition, notice that the probability density function f(y,t) of $Y=X+\sqrt{t}Z$ is the solution of the heat equation $\frac{\partial }{\partial t}f\left(y,t\right)=\frac{1}{2}\frac{\partial^{2}}{\partial y^{2}}f\left(y,t\right)$ with the initial condition that $f\left(y,0\right)=f_{X}\left(y\right)$. Hence, Conjectures 1 and 2, if true, reveal the properties of the differential entropy of functions that satisfy the heat equation. For more results related to diffusion equations, one may refer to [22].

## 6. Conclusions

In this paper, we studied two conjectures on the derivatives of the differential entropy of a general random variable with added Gaussian noise. Regarding the conjecture on the signs of the derivatives made by Cheng and Geng, we introduced the linear matrix inequality approach to provide evidence that their original method might not generalize to orders higher than four. Instead, we considered imposing an additional constraint, namely the log-concavity assumption, and showed the optimality of Gaussian random variables for orders three, four and five. Thus, we made progress on McKean’s conjecture, under a mild condition.

## Appendix A. Proof of Lemma 4

**Proof.** For Equation (26), according to Lemma 1, f(y,t) satisfies the following (heat) equation:

$$\begin{array}{c} f_{t}:=\frac{\partial }{\partial t}f=\frac{1}{2}f_{2}. \end{array}$$

In addition, according to Equation (5),

$$\begin{array}{c} T_{1}=\frac{f_{1}}{f},\;T_{2}=\frac{f_{2}}{f}-\frac{f_{1}^{2}}{f^{2}}. \end{array}$$

Hence,

$$\begin{array}{cc} 2\frac{\partial }{\partial t}T_{0}=2\frac{\partial }{\partial t}\ln f\left(y,t\right) & =\frac{2f_{t}}{f}=\frac{f_{2}}{f}=T_{2}+T_{1}^{2}. \end{array}$$

Now, it follows that, for n≥0,

$$\begin{array}{c} 2\frac{\partial }{\partial t}T_{n}=2\frac{\partial }{\partial t}\left(\frac{\partial^{n}}{\partial y^{n}}T_{0}\right)=\frac{\partial^{n}}{\partial y^{n}}\left(\frac{\partial }{\partial t}2T_{0}\right)=\frac{\partial^{n}}{\partial y^{n}}\left(T_{2}+T_{1}^{2}\right)=T_{n+2}+\sum_{k=0}^{n}C_{n}^{k}T_{k+1}T_{n-k+1}. \end{array}$$

For Equation (27),

$$\begin{array}{c} \frac{d}{dt}E\left[A\right]=\frac{d}{dt}\int fAdy=\int \left(f_{t}A+f\frac{\partial }{\partial t}A\right)dy=\int \left(f\frac{1}{2}\frac{f_{2}}{f}A+f\frac{\partial }{\partial t}A\right)dy=E\left[\frac{1}{2}\left(T_{2}+T_{1}^{2}\right)A+\frac{\partial }{\partial t}A\right]. \end{array}$$

For Equation (28), the derivative is

$$\begin{array}{c} \frac{d}{dt}E\left[T_{n}^{2}\right]\overset{\left(a\right)}{=}\int \left(f\frac{1}{2}\left(T_{2}+T_{1}^{2}\right)T_{n}^{2}+f\frac{\partial }{\partial t}T_{n}^{2}\right)dy=\int \left(f\frac{1}{2}\left(T_{2}+T_{1}^{2}\right)T_{n}^{2}+fT_{n}\times 2\frac{\partial }{\partial t}T_{n}\right)dy, \end{array}$$

where (a) is due to Equation (27). For the first term of the right-hand side, from Lemma 1 and integration by parts,

$$\begin{array}{cc} & \int fT_{n+1}T_{n}T_{1}dy=\int fT_{n}T_{1}dT_{n}=0-\int T_{n}\left(f_{1}T_{n}T_{1}+fT_{n+1}T_{1}+fT_{n}T_{2}\right)dy,\\ \Rightarrow \; & \int fT_{n+1}T_{n}T_{1}dy=-\frac{1}{2}\int fT_{n}^{2}\left(T_{2}+T_{1}^{2}\right)dy. \end{array}$$

For the second term, we have

$$\begin{array}{cc} \int fT_{n}\times 2\frac{\partial }{\partial t}T_{n}dy & \overset{\left(b\right)}{=}\int fT_{n}\left(T_{n+2}+\sum_{k=0}^{n}C_{n}^{k}T_{k+1}T_{n-k+1}\right)dy\\ & =\int fT_{n}\left(2T_{n+1}T_{1}+\sum_{k=1}^{n-1}C_{n}^{k}T_{k+1}T_{n-k+1}\right)dy+\int fT_{n}dT_{n+1}\\ & =\int \left(fT_{n}\left(2T_{n+1}T_{1}+\sum_{k=1}^{n-1}C_{n}^{k}T_{k+1}T_{n-k+1}\right)-T_{n+1}\left(fT_{1}T_{n}+fT_{n+1}\right)\right)dy\\ & =\int f\left(-T_{n+1}^{2}+T_{n+1}T_{n}T_{1}+T_{n}\sum_{k=1}^{n-1}C_{n}^{k}T_{k+1}T_{n-k+1}\right)dy, \end{array}$$

where (b) is due to Equation (26). Combining these two terms together, the third equality is proved. ☐
